# Regulatory B Cell Therapy in Kidney Transplantation

**DOI:** 10.3389/fphar.2021.791450

**Published:** 2021-12-07

**Authors:** Sergio G. Garcia, Noelia Sandoval-Hellín, Marcella Franquesa

**Affiliations:** ^1^ REMAR-IGTP Group, Germans Trias i Pujol Research Institute (IGTP) and Nephrology Department, University Hospital Germans Trias i Pujol (HUGTiP), Can Ruti Campus, Badalona (Barcelona), Catalonia, Spain; ^2^ Department of Cell Biology, Physiology and Immunology, Autonomous University of Barcelona, Bellaterra, Spain

**Keywords:** regulatory B cells, kidney transplantation, graft tolerance, cell therapy, immunosuppressive therapy

## Abstract

In the context of kidney injury, the role of Bregs is gaining interest. In a number of autoimmune diseases, the number and/or the function of Bregs has been shown to be impaired or downregulated, therefore restoring their balance might be a potential therapeutic tool. Moreover, in the context of kidney transplantation their upregulation has been linked to tolerance. However, a specific marker or set of markers that define Bregs as a unique cell subset has not been found and otherwise multiple phenotypes of Bregs have been studied. A quest on the proper markers and induction mechanisms is now the goal of many researchers. Here we summarize the most recent evidence on the role of Bregs in kidney disease by describing the relevance of *in vitro* and *in vivo* Bregs induction as well as the potential use of Bregs as cell therapy agents in kidney transplantation.

## Introduction

B cells are traditionally described to show a primarily effector phenotype: antibody-producing cells with the capacity to present antigen and stimulate T cells through cytokine production (Janeway et al., 1987; Ochsenbein et al., 1999; Harris et al., 2000). However, nowadays it is widely accepted the existence of B cell subsets with regulatory phenotypes (Bregs) involved in suppressing the immune response, inducing tolerance and maintaining homeostasis (Wang et al., 2020). Immunomodulatory functions of Bregs could be mediated by the action of soluble molecules such as IL-10, IL-35, TGF-β or Granzyme B or by cell contact-dependent apoptosis-inducing mechanisms such as PD-L1, FasL or TIGIT (Flores-Borja et al., 2013; Tang et al., 2016; Cai et al., 2019; Chesneau et al., 2020).

Despite essential roles in modulating several diseases, Bregs so far are not known to have a unique or exclusive marker that defines them as a population, but they constitute a heterogeneous cell population that possess a regulatory phenotype and can be found at different stages of B-cell development as reviewed in (Rosser and Mauri, 2015; Oleinika et al., 2019; Long et al., 2021). However, several markers have been proposed as enriched or identifiers of Breg populations. Breg are commonly identified by their expression of IL-10, with transitional phenotypes, CD19^+^CD24^Hi^CD38^Hi^, as the most abundant phenotype in peripheral blood. Nevertheless, since the initial description of IL-10 + Breg, other populations such as plasmablast (CD19^+^CD27^Hi^CD38^+^), regulatory B1 cells (CD19^+^CD25^+^CD71^+^CD73^+^) and memory IL-10^+^ B cells (CD19^+^CD24^Hi^CD27^+^IL-10^+^) have been described as Breg subsets, and different effector molecules have been linked to regulatory B cells identification and function, such as Tim-1, CD5, CD1d, CD25, GMZB, FasL, CD71, etc. as summarized in Table 1.

**TABLE 1:** Breg Human Cellular markers and Effector molecules.

Human regulatory B cell markers		
Breg subsets and molecules	Markers	Reference
Transitional or Immature B10 cells	CD19^+^CD24^Hi^CD38^Hi^IL-10^+^	Blair et al. (2010); Zhang et al. (2012); Flores-Borja et al. (2013); Khoder et al. (2014); Liu et al. (2016)
CD1d^Hi^ B10 B cells	CD19^+^CD1d^Hi^CD5^+^IL-10^+^	Yanaba et al. (2008); Bankoti et al. (2012); Zhang et al. (2012); van der Vlugt et al. (2014); Khan et al. (2015a)
Memory B10 Cells	CD19^+^CD24^Hi^CD27^+^IL-10^+^	Iwata et al. (2011); Salomon et al. (2017); Hasan et al. (2019)
Br1 Cells	CD19^+^CD25^+^CD71^+^CD73^low^	Kubo et al. (2012); Kim et al. (2016)
Plasmablasts	CD19^+^CD27^Hi^CD38^+^	Matsumoto et al. (2014); Shen et al. (2014)
TIM-1^+^ B cells	CD19^+^ TIM-1^+^(TIM-1^+^ B cells present in different B cell subsets)	Ding et al. (2011); Xiao et al. (2012); Aravena et al. (2017); Cherukuri et al. (2021)
GMZB^+^ B cells	CD19^+^GMZB^+^(GMZB^+^ B cells present in different B cell subsets)	Hagn and Jahrsdörfer (2012); Hagn et al. (2012); Lindner et al. (2013); Chesneau et al. (2015); Durand et al. (2015); Zhu et al. (2017)
CD9^+^ B cells	CD19^+^ CD9^+^(CD9^+^ B cells present in different B cell subsets)	Sun et al. (2015); Brosseau et al. (2018a), Brosseau et al. (2018b); Mohd Jaya et al. (2021)
Circulating B cells	CD19^+^CD25^Hi^	Kessel et al. (2012)
	CD27^Hi^CD1d^Hi^CD86^Hi^	
TIGIT^+^ memory B cells	CD24^Hi^ CD27^+^	Hasan et al. (2021)
	CD39^Hi^ IgD^−^IgM^+^CD1c^+^ TIGIT^+^	

Human Regulatory B Cell Effector Molecules		
Breg subsets and molecules	Key Features	Reference
IL-10	Induce Treg, maintain NKT homeostasis, supress effector T cells	Yanaba et al. (2009); Mauri and Bosma (2012); Lykken et al. (2015); Lighaam et al. (2018)
	Modulates plasmacytoid dendritic cells and macrophage and function	
	CD9 and Tim-1 are strongly associated to IL-10 production	
IL-35	Promotes IL-35 and Il-10 production by Treg and Breg	Shen et al. (2014); Wang et al. (2014); Choi and Egwuagu (2021)
	Inhibits pathogenic Th1 and Th17 responses	
PD-L1	Inhibits T cell activation and differentiation by binding PD-1	Khan et al. (2015b); Wang et al. (2019)
FASL	Induces T cell apoptosis by binding FAS	Lundy and Boros (2002); Lundy and Fox (2009); Tang et al. (2016); Wang et al. (2017)
TGF-β	Enhances Treg and Breg induction	Natarajan et al. (2012); Lee et al. (2014)
	Inhibits Th1 differentiation by inhibiting STAT4	
GZMB	Induces T cell apoptosis and strongly suppress T cell proliferation by degradation of the T-cell receptor ζ-chain	Hagn and Jahrsdörfer (2012); Hagn et al. (2012); Lindner et al. (2013); Durand et al. (2015); Zhu et al. (2017); Chesneau et al. (2020)
	Present in several B cell subsets, in peripheral, most commonly found in plasmablasts	
CD73	Supresses effector T cell function by producing adenosine. CD73 catalyses the dephosphorylation of adenine to adenosine	Saze et al. (2013); Kaku et al. (2014)
IDO	Promotes Treg and Breg differentiation	Nouël et al. (2015)

Breg: Regulatory B cell, Br1: Type 1 Regulatory B cell, B10: IL-10-producing regulatory B cells, GZMB: Granzyme B, IDO: indoleamine 2,3-dioxygenase.

The classic conception of the role of B cells in the field of transplantation was called into question in the last decade, following the publication of two simultaneous studies in 2010, highlighting the relevance of B cells in the development of tolerance in renal transplantation. Both studies involved transplant recipients who had developed spontaneous tolerance and stable patients receiving immunosuppressive therapy. The results obtained in “spontaneously tolerant” patients showed the presence of a higher percentage of B cells in peripheral blood, especially naïve and transitional B cells. At the same time, a higher expression of genes involved in B-cell development was also detected in these tolerant patients compared to stable patients on immunosuppressive therapy (Newell et al., 2010; Sagoo et al., 2010).

Since the emergence of these breakthrough results in 2010, the effect of Breg on the development of tolerance has been described several times as reviewed in (Peng et al., 2018; Cherukuri et al., 2021; Long et al., 2021).

The putative tolerance-inducing power of Bregs makes them an interesting target for the development of therapies to combat transplanted kidney rejection. Among the possible treatment strategies that could be considered, two main groups can be distinguished: those aimed at boosting the natural population of Bregs in the donor and, alternatively, therapy based on the transfer of previously expanded or modified Breg *in vitro*. Here we summarize the most recent evidence on the role of Bregs in kidney transplantation by describing the relevance of *in vitro* and *in vivo* induced Breg (iBreg) as well as the potential use of Bregs as cell therapy agents.

## *In Vivo* Bregs Induction

Several therapeutic strategies have been proposed or found to induce Breg *in vitro* and *in vivo* in human patients, both in preclinical and clinical trials, despite the aforementioned difficulty of accurately identify Breg. In the following section we discuss the different drugs and therapies involved in Breg induction *in vivo*.

### Pharmacological Interventions

The use of immunosuppressive regimes combining different drugs has become a staple of clinical transplantation. For the most part, classical immunosuppressive interventions have little to no effect over B cells, and they have shown not to be active inductors of Breg cells with few exceptions.

Starting from classical immunosuppressive regimes, corticosteroids and calcineurin inhibitors (CNI) mildly reduce the number of total naïve and transitional B cells in renal transplant patients, with the exception of tacrolimus having no effect on B cell subsets (Latorre et al., 2016; Rebollo-Mesa et al., 2016; Tebbe et al., 2016; Bottomley et al., 2017).

In patients with IgA vasculitis that had impaired Breg function, the treatment with glucocorticoid prednisolone, promoted an increase in CD5^+^CD1d^+^, CD5^+^CD1d^+^ IL-10^+^, and IL-10^+^ B cell subsets, accompanied by an increase in the serum IL-10 concentration (Hu et al., 2016). However, in Lupus Nephritis patients, same treatment with prednisolone correlated with lower percentages of IL10^+^ B cells (Heinemann et al., 2016).

While low to medium doses of mycophenolate mofetil increase Breg subsets, high doses of mycophenolate reduce both B cell IL-10 and CD80/86 expression on B cells in kidney transplant patients. (Matz et al., 2012; Joly et al., 2014; Rebollo-Mesa et al., 2016; Bottomley et al., 2017).

The effect of mTOR inhibitors over Breg subsets has not been clearly stablished. In kidney transplanted patients sirolimus reduced Transitional B cell populations, while in another report in liver transplant patients, it was described to induce Breg when patients were converted to sirolimus from a tacrolimus based regime (Latorre et al., 2016; Song et al., 2020).

Also the effect of the 6-mercaptopurine analog, Azathioprine, on Bregs has been studied, and it’s know to reduce total, naïve and transitional B cells (Rebollo-Mesa et al., 2016; Bottomley et al., 2017).

The B cell depleting agent rituximab induces rapid depletion of CD20^+^ B cells after administration in a dose-dependent manner, lasting as long as 6 months, followed by a slow recovery (Bergantini et al., 2020). Breg frequencies decrease after administration of the drug, while long term effect of rituximab seems to indirectly stimulate bone marrow to produce transitional B cells when B cells are depleted, coupled with a substantial reduction in CD27^+^ B (memory) cells at long-term follow-up (Moller et al., 2009; Rehnberg et al., 2009).

The costimulation blocker Belatacept has shown promising results in kidney transplanted patients regarding Breg induction. Belatacept increases IL-10 expression and transitional populations, while reducing plasmablast differentiation (Leibler et al., 2014; Xu et al., 2020).

Finally, common induction therapies, such as basiliximab (chimeric anti-CD25) and Thymoglobulin (anti-thymocyte globulin) show no effect over transitional B cells (Longshan et al., 2014; Alfaro et al., 2021) while CAMPATH-1H (anti-CD52) increased transitional B cells and reduced memory B cells (Thompson et al., 2010; Heidt et al., 2012; Cherukuri et al., 2021).

Other immunomodulatory drugs have been described to induce Breg, such as tocilizumab (anti-IL-6) (Assier et al., 2010; Snir et al., 2011), Fingolimod (sphingosine-1-phosphate receptors modulator) (Grützke et al., 2015) and Laquinimod (quinolone-3-carboxiamide) (Toubi et al., 2012).

### Cell and Extracellular Vesicles Therapies

Cell therapies earned a lot of interest as a new approach to induce immunosuppression and tolerance, with an increased presence in clinical trials during the last decade.

Mesenchymal stromal/stem cells (MSC) therapy has been at the forefront of cell therapies in the field of transplantation due to their immunomodulatory and regenerative properties (Pittenger et al., 2019). MSC interact with several cell types, including B cells, inducing regulatory B cells while abrogating plasmablast induction, B cell terminal differentiation and inhibiting antibody production (Comoli et al., 2007; Asari et al., 2009; Guo et al., 2013; Franquesa et al., 2015; Gupte et al., 2017; Perico et al., 2018; Chen et al., 2019, 23). The effect and mechanisms of MSC immunomodulatory action on Bregs has been extensively reviewed (Liu et al., 2020).

A promising alternative to MSC cell therapies are extracellular vesicles (MSC-EVs), reviewed in (Gomzikova et al., 2019; Gowen et al., 2020). EVs emulate parental cell properties, MSC-EVs stimulate tissue regeneration and immune modulation and have been proposed to tackle many diseases including kidney diseases and kidney graft rejection. MSC-EVs have been described *in vitro* to be mediators of Breg induction in a dose dependent manner (Budoni et al., 2013). However, MSC-EV involvement in Breg induction is a complex topic as the EV isolation method used might produce opposite effects as we previously described (Carreras-Planella et al., 2019). Highly purified MSC-EV displayed reduced immunomodulatory capabilities on B cells compared to MSC soluble protein enriched fractions.

In addition to MSC, other cell therapies have been tested and described to induce Breg. Regulatory T cells (Treg) therapy using autologous T cells in kidney transplant patients has been associated with a long-lasting dose-dependent increase of marginal B zone B cells, which are associated with IL-10 production and regulation (Harden et al., 2021). In a different study in a mouse model, CAR-Treg specific to the B cell marker CD19 (Imura et al., 2020) suppressed IgG antibody production and differentiation of B cells in a TGF-β–dependent manner. Regulatory T and B cells work in harmony to stablish homeostasis, and both promote each other induction and expansion as seen in different mouse models (Lee et al., 2014; Wang et al., 2015; Chien and Chiang, 2017) *via* IL-10 and TGF-β.

Tolerogenic Dendritic cells (tolDC) are essential for the induction of Breg in humans and their administration has been described to induce Breg (Boldison et al., 2020). No clinical trials have been performed in kidney transplant patients, but in a phase one safety study in diabetic patients, tolDC increased the frequency of regulatory B cells (Giannoukakis et al., 2011).

### Indirect Intervention: The Role of Microbiota

Previous methods described to induce Bregs focus on tackling the specific either cellular or molecular pathways involved in the maintenance or induction of Breg, but a different approach with growing interest during recent years is to promote balanced stress-free metabolic and immune balance.

In this context, an alternative approach to promote graft tolerance and improve patients’ quality of life, most likely in combination with previous drug interventions and/or cell therapies, would be focus on metabolic interventions by modulation of gut microbiota or other metabolic pathways.

Gut microbiota and dysbiosis are linked to adverse events, reduced quality of life, and an increase of graft rejection in kidney transplanted patients (Lee et al., 2019; Swarte et al., 2020; Pacaud et al., 2021). Gut microbiota interacts with the immune system generating a balance of inflammatory and regulatory responses that maintain the homeostasis with metabolic and immune system effects outside the gut, including the generation of Bregs. B cells have the capability to recognize different bacterial and viral elements by the BCR and TLRs (Gallego-Valle et al., 2018; Mu et al., 2020; Pacaud et al., 2021) and also cytokines and metabolites derived from these microbes, such as short chain fatty acids (SCFAs) (Rosser et al., 2020; Daïen et al., 2021; Pacaud et al., 2021; Zou et al., 2021) expanding Breg subsets. Recent studies have elucidated the role of the SCFA pentanoate in the modulation of mTOR activity, leading to a significant boost of IL-10 production by LPS or CpG stimulated Breg, and a substantial reduction of B cell apoptosis, in addition to reducing expression of IL-17A in effector T cells by inhibiting HDAC (histone deacetylase) activity *via* epigenetic modulation (Luu et al., 2019). In a different study, direct inhibition of HDAC by Entinostat, an HDAC inhibitor, increased IL-10 production by LPS-stimulated B cells. Entinostat activity prevented HDAC binding to the proximal region of the IL-10 expression promoter, increasing binding of NF-κB p65, and enhancing IL-10 expression (Min et al., 2021).

## iBreg Cell Therapy: Is it Feasible

Cell therapy is not a new concept anymore and protocols and clinical trials are being set up to promote tolerance in autoimmune diseases and transplantation in the absence or in a minimized immunosuppressive regime. MSC therapy has taken the lead in this area with several clinical trials already published. In parallel, regulatory immune cell types such as Tregs or tolDCs are the main not-modified immune cell types being studied and used for cell therapy in immune mediated diseases and regenerative approaches. Therefore, the idea of a cell therapy product involving Bregs might sound promising although, to this moment, there are no trials on the use of Breg as a cell therapy. The incomplete knowledge on Breg induction and or expansion, stability, and functional potential and the lack of a consensus Breg signature are just some of the hurdles to be bypassed to generate a safe and efficient cell product. Moreover, we might be dealing with different subsets of Breg depending on the induction cocktail and system used that might present different stability and functionality. We are going in depth on it in the next *In Vitro Breg induction (iBregs)*.

Another matter of concern is the antigen specificity of Breg. Recent studies provide evidence for an essential role of antigen recognition by B cells to generate allograft tolerance in murine models (Kimura et al., 2020; Mohib et al., 2020) and in this line, the critical role of BCR and CD40 expression for Breg development and in transplant models is proven. On the other hand, TGF-β seems to mediate a prominent role in allograft survival (Kimura et al., 2020) while IL-10 essentiality in mediating Breg tolerogenic action is questioned. Insight in the role of Breg antigen specificity may bring the development of chimeric antigen receptor (CAR)-lymphocyte generation to produce cellular therapies with targeted Bregs.

Bregs have also been shown to represent a significant source of serum IgM and IgG during adoptive transfer experiments, and produce antigen-specific, polyreactive and autoreactive antibody specificities (Lo-Man, 2011). However, their role in solid organ transplantation still needs to be defined and new technical advances in nanosciences might bring new opportunities into that area.

The effect that donor or recipient-derived Breg could have in modulating the immune reaction remains unknown if we envision a therapy with autologous or allogeneic Breg in autoimmune diseases or organ transplantation. And as in every cell therapy donor origin (autologous or allogeneic) needs to be carefully considered. The age of the patient appears to be a relevant factor in the capacity of Bregs to produce IL-10 since it is impaired in CD38^Hi^CD24^Hi^ B cells from old individuals (Duggal et al., 2013). Also, autoimmune diseases have been related to disfunctional Bregs where patient’s Breg numbers are normal but they lack the immunomodulatory properties related to this cell “subset” and furthermore, Bregs isolated from patients who had suffered renal graft rejection lost their inhibitory capacity (Nouël et al., 2014). This might be a major problem if we think of autologous cell treatment. There are other mechanistic issues that would have to be addressed such as the time needed to produce enough Breg, infusion timing and dosage, route of administration, and GMP compliance.

### *In Vitro* Breg Induction (iBregs)

The implementation of a reliable and reproducible method of *in vitro* Breg induction and expansion from human B cells is highly required for the development of Breg-based cell therapies. The application of a standardized *in vitro* induction method which generates a well-characterized subset of induced Bregs could compensate for the absence of a human Breg biomarker.

Traditional mechanisms of iBregs induction are mostly based on the stimulation of Toll-like receptors 4 and 9 (TLR4 and TLR9) by bacterial-derived LPS and CpG molecules and the ligation of B-cell antigen receptor (BCR) and CD40 to agonist molecules. In this sense, signaling pathways triggered by TLRs and CD40 accompanied by BCR stimulation might lead to IL-10 production, a conventionally used cytokine for the assessment of *in vitro* Breg induction, *via* activation of transcription factors such as STAT3 (Baba et al., 2015). However, there is also evidence of B cell activation towards effector and/or memory phenotypes when using such strong stimulation methods, and therefore inflammatory cytokines should also be carefully monitored in these induction systems (Lighaam et al., 2018).

Besides IL-10, several groups have described other molecules as key mediators of iBreg regulatory potential. Several “non-classic” Breg markers have been explored, such as the Granzyme B (GZMB) molecule. Currently, the population of GZMB-expressing B cells is represented as a particular subset of Bregs. In addition, a reproducible protocol for the *in vitro* expansion of this cell subset is already reported in the literature (Chesneau et al., 2020).

As we mentioned in *Indirect Intervention: The Role of Microbiota*, the use of SCFAs is being developed in an attempt to mimic the gut microbiota action. This method would be considered an additional novel form of *in vitro* Breg induction as an alternative to boosting the patient’s natural gut microbiota.

Leaving aside the traditional methods and the use of bacterial compounds, our group described the co-culture of tonsil derived-B cells and MSCs from subcutaneous fat as a method of inducing the development of Bregs (Franquesa et al., 2015; Luk et al., 2017).

Common to all the aforementioned methodology, it would be necessary to establish a protocol for the generation of iBregs with a stable phenotype over time. In this regard, the Breg marker CD9 has already been shown to be highly modulated (Mohd Jaya et al., 2021). Therefore, phenotypic and functional stability is a challenge considering the transient nature of some of the Breg phenotypes described (Table 1).

## Discussion

**FIGURE 1:**
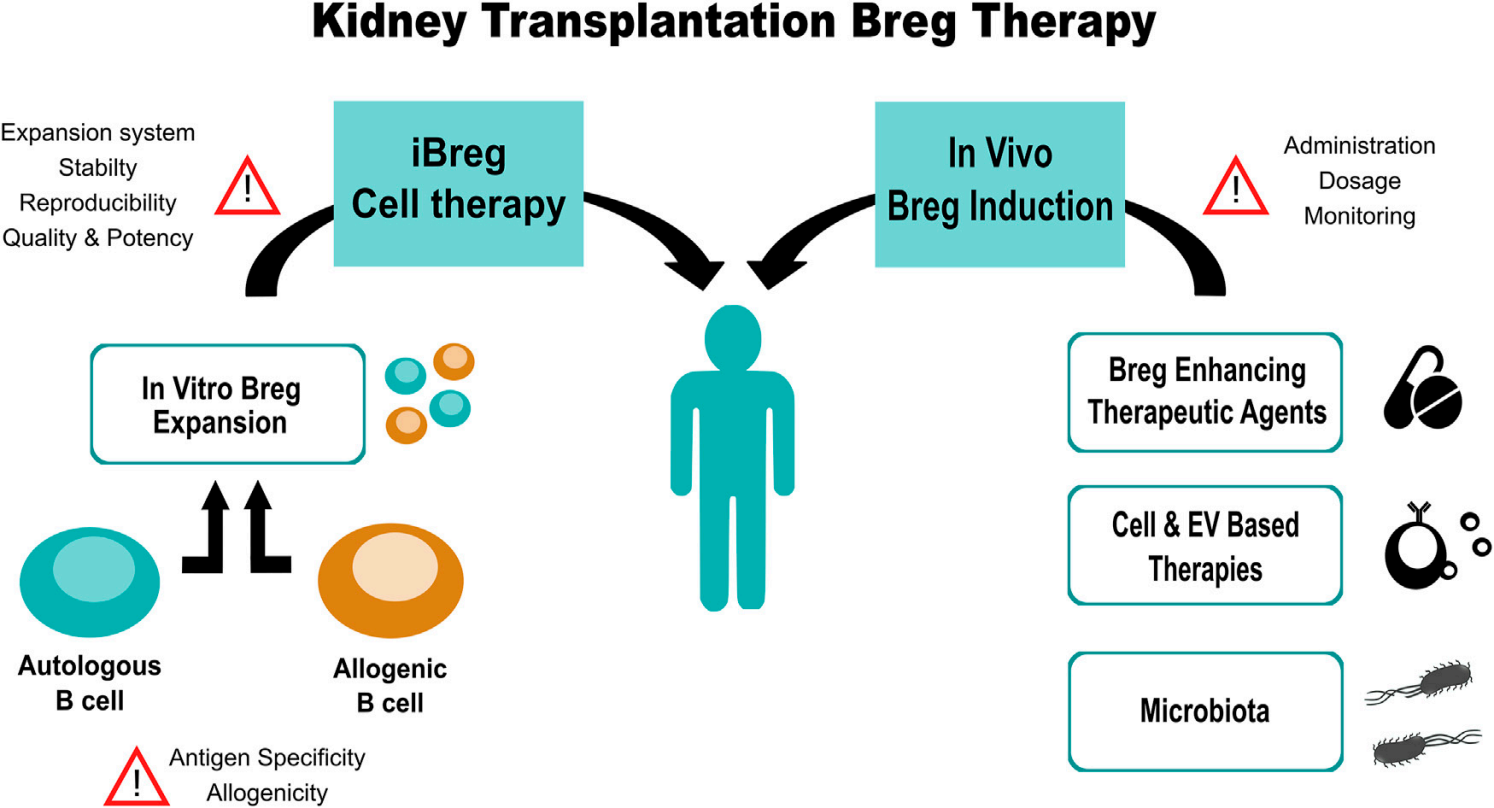
Summarized workflow of Breg-based therapy for kidney transplanted patients, steps and points of concern.

In this mini-review we aimed to summarize the most relevant and recent evidence on the role of Bregs in kidney disease by discussing the relevance of *in vitro* and *in vivo* Breg induction as well as the potential use of Bregs as cell therapy agents (Figure 1). Bregs have been described as major drivers of tolerance in kidney transplantation and in autoimmune diseases. Their upregulated expression has been related to spontaneous tolerant kidney transplant patients, while impaired function and low numbers of Bregs have been associated to several autoimmune diseases.

Breg homeostasis appears to be a cornerstone for immune regulation and tolerance, so several tactics are being approached to reestablish the equilibrium lost in several pathological situations.

One approach is to boost natural Breg populations in patients. Regardless of the limited knowledge of markers, there is already a description of the effect of every immunosuppressant used in kidney transplantation and other anti-inflammatory drugs on Breg populations, allowing for a possible optimization of Breg-induced tolerance. In this line, some immunosuppressants such as Belatacept or Campath, have shown to boost Breg in kidney transplant patients.

Such Breg boosting therapies could be coadjuvant of cellular therapies with MSC or Tregs which have shown capacity to induce Bregs in kidney transplant patients. TolDC have shown similar results in diabetic patients, but have not been tested in clinical trials for kidney transplant. Furthermore, indirect metabolic interventions are slowly gaining track on the field, with increasing publications about the role of gut microbiota and dysbiosis in the maintenance of homeostasis and immune balance, opening new fields of research and clinical research.

On the other hand, Breg application as adoptive cellular therapy is a contentious topic, with no clinical trials on the horizon, due to their difficult identification and expansion. In human, Breg are niche populations in peripheral blood with low expression, with most of them being localized in the spleen, so *in vitro* expansion would be necessary to achieve significant therapeutic effects. The lack of consensus on several key points such as; Breg signature, antigen specificity, alloreactivity, expansion system, stability after administration and dosage, would be critical in the efficacy of such therapy.

In recent years, there has been extensive progress in the understanding of Bregs, many researchers are pursuing the definitive human Breg signature, however this seems rather utopic. Many different markers have been associated to Bregs as reviewed in (Rosser and Mauri, 2015; Shang et al., 2020; Catalán et al., 2021), highlighting the fact that they are found across all B cell subsets.

The differences in phenotype and in secreted factors is not just a make-up signature but it affects Bregs’ mechanism of action, claiming for proper read-outs for the regulatory activity of Bregs. For long time, IL-10 has been the gold-standard to discern between regulatory and non-regulatory B cells, but evidence has shown that, on one hand IL-10 alone is not enough to truly describe Bregs and iBregs, as IL-10 is also an activation marker, and the ratio between inflammatory markers and IL10 (anti-inflammatory cytokine) might be the real hallmark of Bregs. On the other hand, other secreted factors such as GZMB or the expression of FasL or PD-L1 have also been described as key markers of Bregs and iBregs, opening new doors for their mechanistic paths of regulation.

However, the development of improved *in vitro* Bregs induction systems from human B cells, such as the use of SCFAs or MSCs, opens up the possibility of re-educating the patient’s B cells towards a regulatory phenotype and presents a small ray of hope in the context of adoptive Bregs therapy. The ideal *in vitro* induction system should generate, from donor B cells, a sufficient number of iBregs with a stable phenotype and a demonstrable immunodulatory capacity.

## References

[B1] Alfaro R. Legaz I. González-Martínez G. Jimenez-Coll V. Martínez-Banaclocha H. Galián J. A. (2021). Monitoring of B Cell in Kidney Transplantation: Development of a Novel Clusters Analysis and Role of Transitional B Cells in Transplant Outcome. Diagnostics (Basel) 11, 641. 10.3390/diagnostics11040641 33916199 PMC8065535

[B2] Aravena O. Ferrier A. Menon M. Mauri C. Aguillón J. C. Soto L. (2017). TIM-1 Defines a Human Regulatory B Cell Population that Is Altered in Frequency and Function in Systemic Sclerosis Patients. Arthritis Res. Ther. 19, 8. 10.1186/s13075-016-1213-9 28103916 PMC5248463

[B3] Asari S. Itakura S. Ferreri K. Liu C. P. Kuroda Y. Kandeel F. (2009). Mesenchymal Stem Cells Suppress B-Cell Terminal Differentiation. Exp. Hematol. 37, 604–615. 10.1016/j.exphem.2009.01.005 19375651 PMC2747661

[B4] Assier E. Boissier M. C. Dayer J. M. (2010). Interleukin-6: From Identification of the Cytokine to Development of Targeted Treatments. Jt. Bone Spine 77, 532–536. 10.1016/j.jbspin.2010.07.007 20869898

[B5] Baba Y. Matsumoto M. Kurosaki T. (2015). Signals Controlling the Development and Activity of Regulatory B-Lineage Cells. Int. Immunol. 27, 487–493. 10.1093/intimm/dxv027 25957265

[B6] Bankoti R. Gupta K. Levchenko A. Stäger S. (2012). Marginal Zone B Cells Regulate Antigen-specific T Cell Responses during Infection. J. Immunol. 188, 3961–3971. 10.4049/jimmunol.1102880 22412197

[B7] Bergantini L. d'Alessandro M. Cameli P. Vietri L. Vagaggini C. Perrone A. (2020). Effects of Rituximab Therapy on B Cell Differentiation and Depletion. Clin. Rheumatol. 39, 1415–1421. 10.1007/s10067-020-04996-7 32088800

[B8] Blair P. A. Noreña L. Y. Flores-Borja F. Rawlings D. J. Isenberg D. A. Ehrenstein M. R. (2010). CD19(+)CD24(hi)CD38(hi) B Cells Exhibit Regulatory Capacity in Healthy Individuals but Are Functionally Impaired in Systemic Lupus Erythematosus Patients. Immunity 32, 129–140. 10.1016/j.immuni.2009.11.009 20079667

[B9] Boldison J. Da Rosa L. C. Davies J. Wen L. Wong F. S. (2020). Dendritic Cells License Regulatory B Cells to Produce IL-10 and Mediate Suppression of Antigen-specific CD8 T Cells. Cell. Mol. Immunol. 17, 843–855. 10.1038/s41423-019-0324-z 31728048 PMC7395736

[B10] Bottomley M. J. Chen M. Fuggle S. Harden P. N. Wood K. J. (2017). Application of Operational Tolerance Signatures Are Limited by Variability and Type of Immunosuppression in Renal Transplant Recipients: A Cross-Sectional Study. Transpl. Direct 3, e125. 10.1097/TXD.0000000000000638 PMC5361564 28349125

[B11] Brosseau C. Colas L. Magnan A. Brouard S. (2018a). CD9 Tetraspanin: A New Pathway for the Regulation of Inflammation? Front. Immunol. 9, 2316. 10.3389/fimmu.2018.02316 30356731 PMC6189363

[B12] Brosseau C. Durand M. Colas L. Durand E. Foureau A. Cheminant M. A. (2018b). CD9+ Regulatory B Cells Induce T Cell Apoptosis via IL-10 and Are Reduced in Severe Asthmatic Patients. Front. Immunol. 9, 3034. 10.3389/fimmu.2018.03034 30622536 PMC6308143

[B13] Budoni M. Fierabracci A. Luciano R. Petrini S. Di Ciommo V. Muraca M. (2013). The Immunosuppressive Effect of Mesenchymal Stromal Cells on B Lymphocytes Is Mediated by Membrane Vesicles. Cel Transpl. 22, 369–379. 10.3727/096368911X582769 23433427

[B14] Cai X. Zhang L. Wei W. (2019). Regulatory B Cells in Inflammatory Diseases and Tumor. Int. Immunopharmacol. 67, 281–286. 10.1016/j.intimp.2018.12.007 30572252

[B15] Carreras-Planella L. Monguió-Tortajada M. Borràs F. E. Franquesa M. (2019). Immunomodulatory Effect of MSC on B Cells Is Independent of Secreted Extracellular Vesicles. Front. Immunol. 10, 1288. 10.3389/fimmu.2019.01288 31244839 PMC6563675

[B16] Catalán D. Mansilla M. A. Ferrier A. Soto L. Oleinika K. Aguillón J. C. (2021). Immunosuppressive Mechanisms of Regulatory B Cells. Front. Immunol. 12, 611795. 10.3389/fimmu.2021.611795 33995344 PMC8118522

[B17] Chen X. Cai C. Xu D. Liu Q. Zheng S. Liu L. (2019). Human Mesenchymal Stem Cell-Treated Regulatory CD23+CD43+ B Cells Alleviate Intestinal Inflammation. Theranostics 9, 4633–4647. 10.7150/thno.32260 31367246 PMC6643430

[B18] Cherukuri A. Mohib K. Rothstein D. M. (2021). Regulatory B Cells: TIM-1, Transplant Tolerance, and Rejection. Immunol. Rev. 299, 31–44. 10.1111/imr.12933 33484008 PMC7968891

[B19] Chesneau M. Michel L. Dugast E. Chenouard A. Baron D. Pallier A. (2015). Tolerant Kidney Transplant Patients Produce B Cells with Regulatory Properties. J. Am. Soc. Nephrol. 26, 2588–2598. 10.1681/ASN.2014040404 25644114 PMC4587683

[B20] Chesneau M. Mai H. L. Danger R. Le Bot S. Nguyen T. V. Bernard J. (2020). Efficient Expansion of Human Granzyme B-Expressing B Cells with Potent Regulatory Properties. J. Immunol. 205, 2391–2401. 10.4049/jimmunol.2000335 32948686

[B21] Chien C. H. Chiang B. L. (2017). Regulatory T Cells Induced by B Cells: a Novel Subpopulation of Regulatory T Cells. J. Biomed. Sci. 24, 86. 10.1186/s12929-017-0391-3 29151021 PMC5694621

[B22] Choi J. K. Egwuagu C. E. (2021). Interleukin 35 Regulatory B Cells. J. Mol. Biol. 433, 166607. 10.1016/j.jmb.2020.07.019 32755620 PMC7779660

[B23] Comoli P. Ginevri F. Maccario R. Avanzini M. A. Marconi M. Groff A. (2007). Human Mesenchymal Stem Cells Inhibit Antibody Production Induced *In Vitro* by Allostimulation. Nephrol. Dial. Transpl. 23, 1196–1202. 10.1093/ndt/gfm740 18029377

[B24] Daïen C. I. Tan J. Audo R. Mielle J. Quek L. E. Krycer J. R. (2021). Gut-derived Acetate Promotes B10 Cells with Antiinflammatory Effects. JCI Insight 6, e144156. 10.1172/jci.insight.144156 PMC8119207 33729999

[B25] Ding Q. Yeung M. Camirand G. Zeng Q. Akiba H. Yagita H. (2011). Regulatory B Cells Are Identified by Expression of TIM-1 and Can Be Induced through TIM-1 Ligation to Promote Tolerance in Mice. J. Clin. Invest. 121, 3645–3656. 10.1172/JCI46274 21821911 PMC3163958

[B26] Duggal N. A. Upton J. Phillips A. C. Sapey E. Lord J. M. (2013). An Age-Related Numerical and Functional Deficit in CD19(+) CD24(hi) CD38(hi) B Cells Is Associated with an Increase in Systemic Autoimmunity. Aging Cell 12, 873–881. 10.1111/acel.12114 23755918 PMC3814412

[B27] Durand J. Huchet V. Merieau E. Usal C. Chesneau M. Remy S. (2015). Regulatory B Cells with a Partial Defect in CD40 Signaling and Overexpressing Granzyme B Transfer Allograft Tolerance in Rodents. J. Immunol. 195, 5035–5044. 10.4049/jimmunol.1500429 26432892

[B28] Flores-Borja F. Bosma A. Ng D. Reddy V. Ehrenstein M. R. Isenberg D. A. (2013). CD19+CD24hiCD38hi B Cells Maintain Regulatory T Cells while Limiting TH1 and TH17 Differentiation. Sci. Transl. Med. 5, 173ra23. 10.1126/scitranslmed.3005407 23427243

[B29] Franquesa M. Mensah F. K. Huizinga R. Strini T. Boon L. Lombardo E. (2015). Human Adipose Tissue-Derived Mesenchymal Stem Cells Abrogate Plasmablast Formation and Induce Regulatory B Cells Independently of T Helper Cells. Stem Cells 33, 880–891. 10.1002/stem.1881 25376628

[B30] Gallego-Valle J. Pérez-Fernández V. A. Correa-Rocha R. Pion M. (2018). Generation of Human Breg-like Phenotype with Regulatory Function *In Vitro* with Bacteria-Derived Oligodeoxynucleotides. Int. J. Mol. Sci. 19, 1737. 10.3390/ijms19061737 PMC6032322 29895745

[B31] Giannoukakis N. Phillips B. Finegold D. Harnaha J. Trucco M. (2011). Phase I (Safety) Study of Autologous Tolerogenic Dendritic Cells in Type 1 Diabetic Patients. Diabetes Care 34, 2026–2032. 10.2337/dc11-0472 21680720 PMC3161299

[B32] Gomzikova M. O. James V. Rizvanov A. A. (2019). Therapeutic Application of Mesenchymal Stem Cells Derived Extracellular Vesicles for Immunomodulation. Front. Immunol. 10, 2663. 10.3389/fimmu.2019.02663 31849929 PMC6889906

[B33] Gowen A. Shahjin F. Chand S. Odegaard K. E. Yelamanchili S. V. (2020). Mesenchymal Stem Cell-Derived Extracellular Vesicles: Challenges in Clinical Applications. Front. Cel Dev. Biol. 8, 149. 10.3389/fcell.2020.00149 PMC7080981 32226787

[B34] Grützke B. Hucke S. Gross C. C. Herold M. V. Posevitz-Fejfar A. Wildemann B. T. (2015). Fingolimod Treatment Promotes Regulatory Phenotype and Function of B Cells. Ann. Clin. Transl. Neurol. 2, 119–130. 10.1002/acn3.155 25750917 PMC4338953

[B35] Guo Y. Chan K. H. Lai W. H. Siu C. W. Kwan S. C. Tse H. F. (2013). Human Mesenchymal Stem Cells Upregulate CD1dCD5(+) Regulatory B Cells in Experimental Autoimmune Encephalomyelitis. Neuroimmunomodulation 20, 294–303. 10.1159/000351450 23899693

[B36] Gupte K. S. Vanikar A. V. Trivedi H. L. Patel C. N. Patel J. V. (2017). *In-vitro* Generation of Interleukin-10 Secreting B-Regulatory Cells from Donor Adipose Tissue Derived Mesenchymal Stem Cells and Recipient Peripheral Blood Mononuclear Cells for Potential Cell Therapy. Biomed. J. 40, 49–54. 10.1016/j.bj.2017.01.003 28411882 PMC6138595

[B37] Hagn M. Jahrsdörfer B. (2012). Why Do Human B Cells Secrete Granzyme B? Insights into a Novel B-Cell Differentiation Pathway. OncoImmunology 1, 1368–1375. 10.4161/onci.22354 23243600 PMC3518509

[B38] Hagn M. Sontheimer K. Dahlke K. Brueggemann S. Kaltenmeier C. Beyer T. (2012). Human B Cells Differentiate into Granzyme B-Secreting Cytotoxic B Lymphocytes upon Incomplete T-Cell Help. Immunol. Cel Biol. 90, 457–467. 10.1038/icb.2011.64 21808264

[B39] Harden P. N. Game D. S. Sawitzki B. Van der Net J. B. Hester J. Bushell A. (2021). Feasibility, Long-Term Safety, and Immune Monitoring of Regulatory T Cell Therapy in Living Donor Kidney Transplant Recipients. Am. J. Transpl. 21, 1603–1611. 10.1111/ajt.16395 PMC7613119 33171020

[B40] Harris D. P. Haynes L. Sayles P. C. Duso D. K. Eaton S. M. Lepak N. M. (2000). Reciprocal Regulation of Polarized Cytokine Production by Effector B and T Cells. Nat. Immunol. 1, 475–482. 10.1038/82717 11101868

[B41] Hasan M. M. Thompson-Snipes L. Klintmalm G. Demetris A. J. O'Leary J. Oh S. (2019). CD24hiCD38hi and CD24hiCD27+ Human Regulatory B Cells Display Common and Distinct Functional Characteristics. J. Immunol. 203, 2110–2120. 10.4049/jimmunol.1900488 31511354

[B42] Hasan M. M. Nair S. S. O'Leary J. G. Thompson-Snipes L. Nyarige V. Wang J. (2021). Implication of TIGIT+ Human Memory B Cells in Immune Regulation. Nat. Commun. 12, 1534. 10.1038/s41467-021-21413-y 33750787 PMC7943800

[B43] Heidt S. Hester J. Shankar S. Friend P. J. Wood K. J. (2012). B Cell Repopulation after Alemtuzumab Induction-Transient Increase in Transitional B Cells and Long-Term Dominance of Naïve B Cells. Am. J. Transpl. 12, 1784–1792. 10.1111/j.1600-6143.2012.04012.x PMC3387484 22420490

[B44] Heinemann K. Wilde B. Hoerning A. Tebbe B. Kribben A. Witzke O. (2016). Decreased IL-10(+) Regulatory B Cells (Bregs) in Lupus Nephritis Patients. Scand. J. Rheumatol. 45, 312–316. 10.3109/03009742.2015.1126346 26948375

[B45] Hu X. Tai J. Qu Z. Zhao S. Zhang L. Li M. (2016). A Lower Proportion of Regulatory B Cells in Patients with Henoch-Schoenlein Purpura Nephritis. Plos One 11, e0152368. 10.1371/journal.pone.0152368 27030970 PMC4816555

[B46] Imura Y. Ando M. Kondo T. Ito M. Yoshimura A. (2020). CD19-targeted CAR Regulatory T Cells Suppress B Cell Pathology without GvHD. JCI Insight 5, e136185. 10.1172/jci.insight.136185 PMC7453900 32525846

[B47] Iwata Y. Matsushita T. Horikawa M. DiLillo D. J. Yanaba K. Venturi G. M. (2011). Characterization of a Rare IL-10-competent B-Cell Subset in Humans that Parallels Mouse Regulatory B10 Cells. Blood 117, 530–541. 10.1182/blood-2010-07-294249 20962324 PMC3031478

[B48] Janeway C. A. Ron J. Katz M. E. (1987). The B Cell Is the Initiating Antigen-Presenting Cell in Peripheral Lymph Nodes. J. Immunol. 138, 1051–1055. 3100626

[B49] Joly M. S. Martin R. P. Mitra-Kaushik S. Phillips L. D'Angona A. Richards S. M. (2014). Transient Low-Dose Methotrexate Generates B Regulatory Cells that Mediate Antigen-specific Tolerance to Alglucosidase Alfa. J. Immunol. 193, 3947–3958. 10.4049/jimmunol.1303326 25210119

[B50] Kaku H. Cheng K. F. Al-Abed Y. Rothstein T. L. (2014). A Novel Mechanism of B Cell-Mediated Immune Suppression through CD73 Expression and Adenosine Production. J. Immunol. 193, 5904–5913. 10.4049/jimmunol.1400336 25392527 PMC4321875

[B51] Kessel A. Haj T. Peri R. Snir A. Melamed D. Sabo E. (2012). Human CD19(+)CD25(high) B Regulatory Cells Suppress Proliferation of CD4(+) T Cells and Enhance Foxp3 and CTLA-4 Expression in T-Regulatory Cells. Autoimmun. Rev. 11, 670–677. 10.1016/j.autrev.2011.11.018 22155204

[B52] Khan A. R. Amu S. Saunders S. P. Hams E. Blackshields G. Leonard M. O. (2015a). Ligation of TLR7 on CD19+CD1dhiB Cells Suppresses Allergic Lung Inflammation via Regulatory T Cells. Eur. J. Immunol. 45, 1842–1854. 10.1002/eji.201445211 25763771

[B53] Khan A. R. Hams E. Floudas A. Sparwasser T. Weaver C. T. Fallon P. G. (2015b). PD-L1hi B Cells Are Critical Regulators of Humoral Immunity. Nat. Commun. 6, 5997. 10.1038/ncomms6997 25609381

[B54] Khoder A. Sarvaria A. Alsuliman A. Chew C. Sekine T. Cooper N. (2014). Regulatory B Cells Are Enriched within the IgM Memory and Transitional Subsets in Healthy Donors but Are Deficient in Chronic GVHD. Blood 124, 2034–2045. 10.1182/blood-2014-04-571125 25051962 PMC4186534

[B55] Kim A. S. Doherty T. A. Karta M. R. Das S. Baum R. Rosenthal P. (2016). Regulatory B Cells and T Follicular Helper Cells Are Reduced in Allergic Rhinitis. J. Allergy Clin. Immunol. 138, 1192. 10.1016/j.jaci.2016.03.017 27142393 PMC5053844

[B56] Kimura S. Rickert C. G. Kojima L. Aburawi M. Tanimine N. Fontan F. (2020). Regulatory B Cells Require Antigen Recognition for Effective Allograft Tolerance Induction. Am. J. Transpl. 20, 977–987. 10.1111/ajt.15739 PMC7372932 31823520

[B57] Kubo S. Yamada T. Osawa Y. Ito Y. Narita N. Fujieda S. (2012). Cytosine-phosphate-guanosine-DNA Induces CD274 Expression in Human B Cells and Suppresses T Helper Type 2 Cytokine Production in Pollen Antigen-Stimulated CD4-Positive Cells. Clin. Exp. Immunol. 169, 1–9. 10.1111/j.1365-2249.2012.04585.x 22670772 PMC3390467

[B58] Latorre I. Esteve-Sole A. Redondo D. Giest S. Argilaguet J. Alvarez S. (2016). Calcineurin and mTOR Inhibitors Have Opposing Effects on Regulatory T Cells while Reducing Regulatory B Cell Populations in Kidney Transplant Recipients. Transpl. Immunol. 35, 1–6. 10.1016/j.trim.2016.01.004 26836476

[B59] Lee K. M. Stott R. T. Zhao G. SooHoo J. Xiong W. Lian M. M. (2014). TGF-β-producing Regulatory B Cells Induce Regulatory T Cells and Promote Transplantation Tolerance. Eur. J. Immunol. 44, 1728–1736. 10.1002/eji.201344062 24700192 PMC4048633

[B60] Lee J. R. Magruder M. Zhang L. Westblade L. F. Satlin M. J. Robertson A. (2019). Gut Microbiota Dysbiosis and Diarrhea in Kidney Transplant Recipients. Am. J. Transpl. 19, 488–500. 10.1111/ajt.14974 PMC6301138 29920927

[B61] Leibler C. Matignon M. Pilon C. Montespan F. Bigot J. Lang P. (2014). Kidney Transplant Recipients Treated with Belatacept Exhibit Increased Naïve and Transitional B Cells. Am. J. Transpl. 14, 1173–1182. 10.1111/ajt.12721 24730563

[B62] Lighaam L. C. Unger P. A. Vredevoogd D. W. Verhoeven D. Vermeulen E. Turksma A. W. (2018). In Vitro-Induced Human IL-10+ B Cells Do Not Show a Subset-Defining Marker Signature and Plastically Co-express IL-10 with Pro-inflammatory Cytokines. Front. Immunol. 9, 1913. 10.3389/fimmu.2018.01913 30258433 PMC6143818

[B63] Lindner S. Dahlke K. Sontheimer K. Hagn M. Kaltenmeier C. Barth T. F. (2013). Interleukin 21-induced Granzyme B-Expressing B Cells Infiltrate Tumors and Regulate T Cells. Cancer Res. 73, 2468–2479. 10.1158/0008-5472.CAN-12-3450 23384943

[B64] Liu Y. Cheng L. S. Wu S. D. Wang S. Q. Li L. She W. M. (2016). IL-10-producing Regulatory B-Cells Suppressed Effector T-Cells but Enhanced Regulatory T-Cells in Chronic HBV Infection. Clin. Sci. (Lond) 130, 907–919. 10.1042/CS20160069 26980345

[B65] Liu J. Liu Q. Chen X. (2020). The Immunomodulatory Effects of Mesenchymal Stem Cells on Regulatory B Cells. Front. Immunol. 11, 1843. 10.3389/fimmu.2020.01843 32922398 PMC7456948

[B66] Lo-Man R. (2011). Regulatory B Cells Control Dendritic Cell Functions. Immunotherapy 3, 19–20. 10.2217/imt.11.34 21524162

[B67] Long W. Zhang H. Yuan W. Lan G. Lin Z. Peng L. (2021). The Role of Regulatory B Cells in Kidney Diseases. Front. Immunol. 12, 683926. 10.3389/fimmu.2021.683926 34108975 PMC8183681

[B68] Longshan L. Dongwei L. Qian F. Jun L. Suxiong D. Yitao Z. (2014). Dynamic Analysis of B-Cell Subsets in De Novo Living Related Kidney Transplantation with Induction Therapy of Basiliximab. Transpl. Proc. 46, 363–367. 10.1016/j.transproceed.2013.12.033 24655964

[B69] Luk F. Carreras-Planella L. Korevaar S. S. de Witte S. F. H. Borràs F. E. Betjes M. G. H. (2017). Inflammatory Conditions Dictate the Effect of Mesenchymal Stem or Stromal Cells on B Cell Function. Front. Immunol. 8, 1042. 10.3389/fimmu.2017.01042 28894451 PMC5581385

[B70] Lundy S. K. Boros D. L. (2002). Fas Ligand-Expressing B-1a Lymphocytes Mediate CD4(+)-T-Cell Apoptosis during Schistosomal Infection: Induction by Interleukin 4 (IL-4) and IL-10. Infect. Immun. 70, 812–819. 10.1128/IAI.70.2.812-819.2002 11796615 PMC127725

[B71] Lundy S. K. Fox D. A. (2009). Reduced Fas Ligand-Expressing Splenic CD5+ B Lymphocytes in Severe Collagen-Induced Arthritis. Arthritis Res. Ther. 11, R128. 10.1186/ar2795 19706160 PMC2745812

[B72] Luu M. Pautz S. Kohl V. Singh R. Romero R. Lucas S. (2019). The Short-Chain Fatty Acid Pentanoate Suppresses Autoimmunity by Modulating the Metabolic-Epigenetic Crosstalk in Lymphocytes. Nat. Commun. 10, 760. 10.1038/s41467-019-08711-2 30770822 PMC6377655

[B73] Lykken J. M. Candando K. M. Tedder T. F. (2015). Regulatory B10 Cell Development and Function. Int. Immunol. 27, 471–477. 10.1093/intimm/dxv046 26254185 PMC4817073

[B74] Matsumoto M. Baba A. Yokota T. Nishikawa H. Ohkawa Y. Kayama H. (2014). Interleukin-10-Producing Plasmablasts Exert Regulatory Function in Autoimmune Inflammation. Immunity 41, 1040–1051. 10.1016/j.immuni.2014.10.016 25484301

[B75] Matz M. Lehnert M. Lorkowski C. Fabritius K. Unterwalder N. Doueiri S. (2012). Effects of Sotrastaurin, Mycophenolic Acid and Everolimus on Human B-Lymphocyte Function and Activation. Transpl. Int. 25, 1106–1116. 10.1111/j.1432-2277.2012.01537.x 22816666

[B76] Mauri C. Bosma A. (2012). Immune Regulatory Function of B Cells. Annu. Rev. Immunol. 30, 221–241. 10.1146/annurev-immunol-020711-074934 22224776

[B77] Min K. Y. Lee M. B. Hong S. H. Lee D. Jo M. G. Lee J. E. (2021). Entinostat, a Histone Deacetylase Inhibitor, Increases the Population of IL-10+ Regulatory B Cells to Suppress Contact Hypersensitivity. BMB Rep. 54, 534–539. 10.5483/BMBRep.2021.54.10.092 34488930 PMC8560462

[B78] Mohd Jaya F. N. Garcia S. G. Borras F. E. Guerrero D. Chan G. C. F. Franquesa M. (2021). *In Vitro* Characterization of Human CD24hiCD38hi Regulatory B Cells Shows CD9 Is Not a Stable Breg Cell Marker. Int. J. Mol. Sci. 22, 4583. 10.3390/ijms22094583 33925530 PMC8123770

[B79] Mohib K. Cherukuri A. Zhou Y. Ding Q. Watkins S. C. Rothstein D. M. (2020). Antigen-dependent Interactions between Regulatory B Cells and T Cells at the T:B Border Inhibit Subsequent T Cell Interactions with DCs. Am. J. Transpl. 20, 52–63. 10.1111/ajt.15546 PMC8117747 31355483

[B80] Möller B. Aeberli D. Eggli S. Fuhrer M. Vajtai I. Vögelin E. (2009). Class-switched B Cells Display Response to Therapeutic B-Cell Depletion in Rheumatoid Arthritis. Arthritis Res. Ther. 11, R62. 10.1186/ar2686 19419560 PMC2714106

[B81] Mu Q. Edwards M. R. Swartwout B. K. Cabana Puig X. Mao J. Zhu J. (2020). Gut Microbiota and Bacterial DNA Suppress Autoimmunity by Stimulating Regulatory B Cells in a Murine Model of Lupus. Front. Immunol. 11, 593353. 10.3389/fimmu.2020.593353 33240280 PMC7683516

[B82] Natarajan P. Singh A. McNamara J. T. Secor E. R. Guernsey L. A. Thrall R. S. (2012). Regulatory B Cells from Hilar Lymph Nodes of Tolerant Mice in a Murine Model of Allergic Airway Disease Are CD5+, Express TGF-β, and Co-localize with CD4+Foxp3+ T Cells. Mucosal Immunol. 5, 691–701. 10.1038/mi.2012.42 22718263 PMC3480990

[B83] Newell K. A. Asare A. Kirk A. D. Gisler T. D. Bourcier K. Suthanthiran M. (2010). Identification of a B Cell Signature Associated with Renal Transplant Tolerance in Humans. J. Clin. Invest. 120, 1836–1847. 10.1172/JCI39933 20501946 PMC2877933

[B84] Nouël A. Ségalen I. Jamin C. Doucet L. Caillard S. Renaudineau Y. (2014). B Cells Display an Abnormal Distribution and an Impaired Suppressive Function in Patients with Chronic Antibody-Mediated Rejection. Kidney Int. 85, 590–599. 10.1038/ki.2013.457 24284517

[B85] Nouël A. Pochard P. Simon Q. Ségalen I. Le Meur Y. Pers J. O. (2015). B-Cells Induce Regulatory T Cells through TGF-β/IDO Production in A CTLA-4 Dependent Manner. J. Autoimmun. 59, 53–60. 10.1016/j.jaut.2015.02.004 25753821

[B86] Ochsenbein A. F. Fehr T. Lutz C. Suter M. Brombacher F. Hengartner H. (1999). Control of Early Viral and Bacterial Distribution and Disease by Natural Antibodies. Science 286, 2156–2159. 10.1126/science.286.5447.2156 10591647

[B87] Oleinika K. Mauri C. Salama A. D. (2019). Effector and Regulatory B Cells in Immune-Mediated Kidney Disease. Nat. Rev. Nephrol. 15, 11–26. 10.1038/s41581-018-0074-7 30443016

[B88] Pacaud M. Colas L. Brouard S. (2021). Microbiota and Immunoregulation: A Focus on Regulatory B Lymphocytes and Transplantation. Am. J. Transpl. 21, 2341–2347. 10.1111/ajt.16522 33559282

[B89] Peng B. Ming Y. Yang C. (2018). Regulatory B Cells: the Cutting Edge of Immune Tolerance in Kidney Transplantation. Cell Death Dis. 9, 109. 10.1038/s41419-017-0152-y 29371592 PMC5833552

[B90] Perico N. Casiraghi F. Todeschini M. Cortinovis M. Gotti E. Portalupi V. (2018). Long-Term Clinical and Immunological Profile of Kidney Transplant Patients Given Mesenchymal Stromal Cell Immunotherapy. Front. Immunol. 9, 1359. 10.3389/fimmu.2018.01359 29963053 PMC6014158

[B91] Pittenger M. F. Discher D. E. Péault B. M. Phinney D. G. Hare J. M. Caplan A. I. (2019). Mesenchymal Stem Cell Perspective: Cell Biology to Clinical Progress. Npj Regen. Med. 4, 22. 10.1038/s41536-019-0083-6 31815001 PMC6889290

[B92] Rebollo-Mesa I. Nova-Lamperti E. Mobillo P. Runglall M. Christakoudi S. Norris S. (2016). Biomarkers of Tolerance in Kidney Transplantation: Are We Predicting Tolerance or Response to Immunosuppressive Treatment? Am. J. Transpl. 16, 3443–3457. 10.1111/ajt.13932 PMC5132071 27328267

[B93] Rehnberg M. Amu S. Tarkowski A. Bokarewa M. I. Brisslert M. (2009). Short- and Long-Term Effects of Anti-CD20 Treatment on B Cell Ontogeny in Bone Marrow of Patients with Rheumatoid Arthritis. Arthritis Res. Ther. 11, R123. 10.1186/ar2789 19686595 PMC2745807

[B94] Rosser E. C. Mauri C. (2015). Regulatory B Cells: Origin, Phenotype, and Function. Immunity 42, 607–612. 10.1016/j.immuni.2015.04.005 25902480

[B95] Rosser E. C. Piper C. J. M. Matei D. E. Blair P. A. Rendeiro A. F. Orford M. (2020). Microbiota-Derived Metabolites Suppress Arthritis by Amplifying Aryl-Hydrocarbon Receptor Activation in Regulatory B Cells. Cell Metab. 31, 837. 10.1016/j.cmet.2020.03.003 32213346 PMC7156916

[B96] Sagoo P. Perucha E. Sawitzki B. Tomiuk S. Stephens D. A. Miqueu P. (2010). Development of a Cross-Platform Biomarker Signature to Detect Renal Transplant Tolerance in Humans. J. Clin. Invest. 120, 1848–1861. 10.1172/JCI39922 20501943 PMC2877932

[B97] Salomon S. Guignant C. Morel P. Flahaut G. Brault C. Gourguechon C. (2017). Th17 and CD24hiCD27+ Regulatory B Lymphocytes Are Biomarkers of Response to Biologics in Rheumatoid Arthritis. Arthritis Res. Ther. 19, 33. 10.1186/s13075-017-1244-x 28183330 PMC5301325

[B98] Saze Z. Schuler P. J. Hong C. S. Cheng D. Jackson E. K. Whiteside T. L. (2013). Adenosine Production by Human B Cells and B Cell-Mediated Suppression of Activated T Cells. Blood 122, 9–18. 10.1182/blood-2013-02-482406 23678003 PMC3701906

[B99] Shang J. Zha H. Sun Y. (2020). Phenotypes, Functions, and Clinical Relevance of Regulatory B Cells in Cancer. Front. Immunol. 11, 582657. 10.3389/fimmu.2020.582657 33193391 PMC7649814

[B100] Shen P. Roch T. Lampropoulou V. O'Connor R. A. Stervbo U. Hilgenberg E. (2014). IL-35-producing B Cells Are Critical Regulators of Immunity during Autoimmune and Infectious Diseases. Nature 507, 366–370. 10.1038/nature12979 24572363 PMC4260166

[B101] Snir A. Kessel A. Haj T. Rosner I. Slobodin G. Toubi E. (2011). Anti-IL-6 Receptor Antibody (Tocilizumab): a B Cell Targeting Therapy. Clin. Exp. Rheumatol. 29, 697–700. 10.1136/ard.2010.149005.1 21813064

[B102] Song J. Du G. Chen W. Bao P. Li B. Lu Q. (2020). The Advantage of Sirolimus in Amplifying Regulatory B Cells and Regulatory T Cells in Liver Transplant Patients. Eur. J. Pharmacol. 869, 172872. 10.1016/j.ejphar.2019.172872 31846626

[B103] Sun J. Wang J. Pefanis E. Chao J. Rothschild G. Tachibana I. (2015). Transcriptomics Identify CD9 as a Marker of Murine IL-10-Competent Regulatory B Cells. Cell Rep. 13, 1110–1117. 10.1016/j.celrep.2015.09.070 26527007 PMC4644501

[B104] Swarte J. C. Douwes R. M. Hu S. Vich Vila A. Eisenga M. F. van Londen M. (2020). Characteristics and Dysbiosis of the Gut Microbiome in Renal Transplant Recipients. J. Clin. Med. 9, 386. 10.3390/jcm9020386 PMC7074359 32024079

[B105] Tang Y. Jiang Q. Ou Y. Zhang F. Qing K. Sun Y. (2016). BIP Induces Mice CD19(hi) Regulatory B Cells Producing IL-10 and Highly Expressing PD-L1, FasL. Mol. Immunol. 69, 44–51. 10.1016/j.molimm.2015.10.017 26655428

[B106] Tebbe B. Wilde B. Ye Z. Wang J. Wang X. Jian F. (2016). Renal Transplant Recipients Treated with Calcineurin-Inhibitors Lack Circulating Immature Transitional CD19+CD24hiCD38hi Regulatory B-Lymphocytes. Plos One 11, e0153170. 10.1371/journal.pone.0153170 27045291 PMC4821620

[B107] Thompson S. A. Jones J. L. Cox A. L. Compston D. A. Coles A. J. (2010). B-cell Reconstitution and BAFF after Alemtuzumab (Campath-1H) Treatment of Multiple Sclerosis. J. Clin. Immunol. 30, 99–105. 10.1007/s10875-009-9327-3 19763798

[B108] Toubi E. Nussbaum S. Staun-Ram E. Snir A. Melamed D. Hayardeny L. (2012). Laquinimod Modulates B Cells and Their Regulatory Effects on T Cells in Multiple Sclerosis. J. Neuroimmunol. 251, 45–54. 10.1016/j.jneuroim.2012.07.003 22846497

[B109] van der Vlugt L. E. Zinsou J. F. Ozir-Fazalalikhan A. Kremsner P. G. Yazdanbakhsh M. Adegnika A. A. (2014). Interleukin 10 (IL-10)-producing CD1dhi Regulatory B Cells from Schistosoma Haematobium-Infected Individuals Induce IL-10-positive T Cells and Suppress Effector T-Cell Cytokines. J. Infect. Dis. 210, 1207–1216. 10.1093/infdis/jiu257 24795476

[B110] Wang R. X. Yu C. R. Dambuza I. M. Mahdi R. M. Dolinska M. B. Sergeev Y. V. (2014). Interleukin-35 Induces Regulatory B Cells that Suppress Autoimmune Disease. Nat. Med. 20, 633–641. 10.1038/nm.3554 24743305 PMC4048323

[B111] Wang L. Ray A. Jiang X. Wang J. Y. Basu S. Liu X. (2015). T Regulatory Cells and B Cells Cooperate to Form a Regulatory Loop that Maintains Gut Homeostasis and Suppresses Dextran Sulfate Sodium-Induced Colitis. Mucosal Immunol. 8, 1297–1312. 10.1038/mi.2015.20 25807185 PMC4583327

[B112] Wang K. Tao L. Su J. Zhang Y. Zou B. Wang Y. (2017). TLR4 Supports the Expansion of FasL+CD5+CD1dhi Regulatory B Cells, Which Decreases in Contact Hypersensitivity. Mol. Immunol. 87, 188–199. 10.1016/j.molimm.2017.04.016 28505514

[B113] Wang X. Wang G. Wang Z. Liu B. Han N. Li J. (2019). PD-1-expressing B Cells Suppress CD4+ and CD8+ T Cells via PD-1/PD-L1-dependent Pathway. Mol. Immunol. 109, 20–26. 10.1016/j.molimm.2019.02.009 30851633

[B114] Wang L. Fu Y. Chu Y. (2020). Regulatory B Cells. Adv. Exp. Med. Biol. 1254, 87–103. 10.1007/978-981-15-3532-1_8 32323272

[B115] Xiao S. Brooks C. R. Zhu C. Wu C. Sweere J. M. Petecka S. (2012). Defect in Regulatory B-Cell Function and Development of Systemic Autoimmunity in T-Cell Ig Mucin 1 (Tim-1) Mucin Domain-Mutant Mice. Proc. Natl. Acad. Sci. U S A. 109, 12105–12110. 10.1073/pnas.1120914109 22773818 PMC3409739

[B116] Xu H. Mehta A. K. Gao Q. Lee H. J. Ghali A. Guasch A. (2020). B Cell Reconstitution Following Alemtuzumab Induction under a Belatacept-Based Maintenance Regimen. Am. J. Transpl. 20, 653–662. 10.1111/ajt.15639 PMC7202689 31596034

[B117] Yanaba K. Bouaziz J. D. Haas K. M. Poe J. C. Fujimoto M. Tedder T. F. (2008). A Regulatory B Cell Subset with a Unique CD1dhiCD5+ Phenotype Controls T Cell-dependent Inflammatory Responses. Immunity 28, 639–650. 10.1016/j.immuni.2008.03.017 18482568

[B118] Yanaba K. Bouaziz J. D. Matsushita T. Tsubata T. Tedder T. F. (2009). The Development and Function of Regulatory B Cells Expressing IL-10 (B10 Cells) Requires Antigen Receptor Diversity and TLR Signals. J. Immunol. 182, 7459–7472. 10.4049/jimmunol.0900270 19494269 PMC3733128

[B119] Zhang M. Zheng X. Zhang J. Zhu Y. Zhu X. Liu H. (2012). CD19(+)CD1d(+)CD5(+) B Cell Frequencies Are Increased in Patients with Tuberculosis and Suppress Th17 Responses. Cell. Immunol. 274, 89–97. 10.1016/j.cellimm.2012.01.007 22361174

[B120] Zhu J. Zeng Y. Dolff S. Bienholz A. Lindemann M. Brinkhoff A. (2017). Granzyme B Producing B-Cells in Renal Transplant Patients. Clin. Immunol. 184, 48–53. 10.1016/j.clim.2017.04.016 28461110

[B121] Zou F. Qiu Y. Huang Y. Zou H. Cheng X. Niu Q. (2021). Effects of Short-Chain Fatty Acids in Inhibiting HDAC and Activating P38 MAPK Are Critical for Promoting B10 Cell Generation and Function. Cel Death Dis 12, 582. 10.1038/s41419-021-03880-9 PMC8184914 34099635

